# Cytotoxic Effects of 2-Bromopropane on Embryonic Development in Mouse Blastocysts

**DOI:** 10.3390/ijms11020731

**Published:** 2010-02-11

**Authors:** Wen-Hsiung Chan

**Affiliations:** Department of Bioscience Technology and Center for Nanotechnology, Chung Yuan Christian University, Chung Li 32023, Taiwan; E-Mail: whchan@cycu.edu.tw; Tel.: +886-3-2653515; Fax: +886-3-2653599

**Keywords:** 2-bromopropane, blastocyst, apoptosis, embryonic development

## Abstract

2-Bromopropane (2-BP), an alternative to ozone-depleting solvents, is used as a cleaning solvent. Here, we examined the cytotoxic effects of 2-bromopropane (2-BP) on mouse embryos at the blastocyst stage, subsequent embryonic attachment and outgrowth *in vitro*, and *in vivo* implantation via embryo transfer. Mouse blastocysts were incubated in medium with or without 2-BP (2.5, 5 or 10 μM) for 24 h. Cell proliferation and growth were investigated with dual differential staining, apoptosis was analyzed by terminal deoxynucleotidyl transferase-mediated dUTP nick-end labeling (TUNEL) analysis, and implantation and post-implantation development of embryos were assessed using *in vitro* development analysis and *in vivo* embryo transfer, respectively. Blastocysts treated with 5 or 10 μM 2-BP displayed significantly increased apoptosis, and decreased inner cell mass (ICM) and trophectoderm (TE) cell number. Additionally, the implantation success rates of 2-BP-pretreated blastocysts were lower than those of untreated controls. *In vitro* treatment with 5 or 10 μM 2-BP was associated with increased resorption of postimplantation embryos, and decreased placental and fetal weights. Our results collectively indicate that *in vitro* exposure to 2-BP induces apoptosis, suppresses implantation rates after transfer to host mice, and retards early postimplantation development.

## 1. Introduction

2-Bromopropane (2-BP) is used as a cleaning solvent and as an alternative to ozone-depleting solvents. Previous studies report a high incidence of oligozoospermia in male workers after long-term exposure to 2-BP [1–3]. Several animal studies further validate the potential injury effects of 2-BP on reproductive, hematopoietic, central nervous, and immune systems [4–11]. In cytotoxicity experiments, mouse embryos treated with 2-BP displayed micronuclei formation and decreased embryo cell number [12]. Moreover, 2-BP was recently identified as a potent DNA damaging agent [5,8]. These results collectively suggest that 2-BP induces various toxicities via activity as a DNA damaging agent. A reproductive toxicity investigation further demonstrated that exposure to 2-BP induced testicular or ovarian dysfunction, causing injury to early types of spermatogenic cells or primordial follicles and oocytes of rats [4,6]. Furthermore, experiments investigating the effects of 2-BP on pre- and postnatal development showed that exposure of pregnant or lactating female rats to 2-BP resulted in delivery rate decrease, peri- and postnatal death increase, loss of body weight development, and increased incidence of reproductive organ dysfunction [13]. However, the regulatory mechanisms underlying the potential adverse effects of 2-BP on embryo-fetal development are yet to be established.

Apoptosis plays an important role in development and disease [14]. A number of studies demonstrate that apoptosis functions in normal embryonic development [15–17]. Conversely, chemical teratogens induce excessive apoptosis in early embryos, leading to developmental injury [18–22]. We recently showed that some natural chemical compounds and mycotoxin induce cellular apoptosis and cytotoxicity in mouse blastocysts [20,23–28]. Clearly, chemical teratogen treatment of mouse blastocysts induces apoptosis, decreases cell numbers, retards early postimplantation blastocyst development, and increases early-stage blastocyst death *in vitro*, while dietary chemical compounds appear to negatively affect mouse embryonic development *in vivo* by triggering apoptosis and inhibiting proliferation.

In the present study, we examine the cytotoxic effects of 2-BP on mouse blastocysts and related regulatory mechanisms. In our experiments, 2-BP suppressed embryonic cell proliferation during the blastocyst stage, largely by inducing apoptosis in the inner cell mass (ICM) and trophectoderm (TE). We additionally monitored subsequent developmental injury of blastocysts *in vitro* and following implantation *in vivo* via embryo transfer.

## 2. Results and Discussion

**Figure 1.**
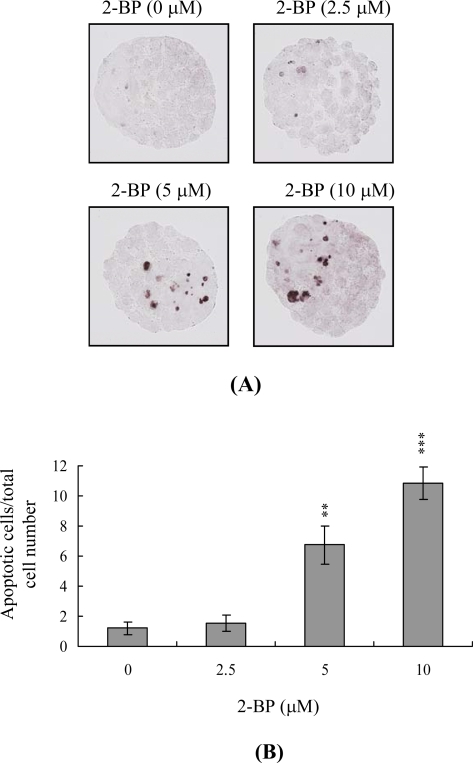
2-BP induces apoptosis in mouse blastocysts. (A) Mouse blastocysts were treated with or without 2-BP (2.5, 5 or 10 μM) for 24 h and apoptosis was examined by TUNEL staining. The results were visualized by light microscopy, which shows TUNEL-positive cells in black. (B) The mean number of apoptotic (TUNEL-positive) cells per total cell number was calculated. Values are presented as means ± SD of five to eight determinations. **P < 0.01 and ***P < 0.001 *vs.* the control group.

Mouse blastocysts were treated with 2.5, 5 or 10 μM 2-BP at 37 °C for 24 h or left untreated, and apoptosis was monitored using the TUNEL method. We observed a concentration-dependent increase in apoptosis in blastocysts treated with 2-BP (5 and 10 μM) (Figure 1A). Quantitative analysis disclosed 6.7- to 10.9-fold higher levels of apoptotic cells in 2-BP-treated blastocysts *vs.* untreated controls (Figure 1B). Our results clearly indicate that 2-BP induces apoptosis in mouse blastocysts.

**Figure 2.**
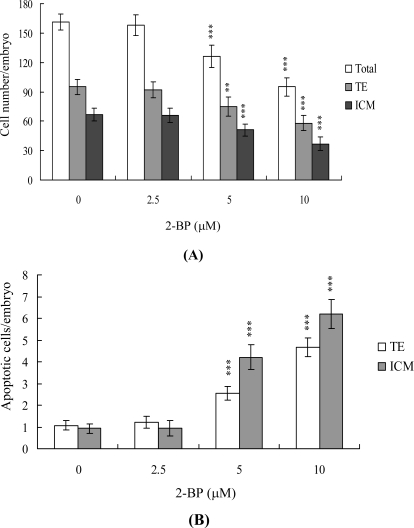
Effects of 2-BP on blastocyst viability. Mouse blastocysts were treated with or without 2-BP (2.5, 5 or 10 μM) for 24 h. (A) The total number of cells per blastocyst and the cell numbers in the inner cell mass (ICM) and trophectoderm (TE) were counted. (B) The Annexin V-positive/PI-negative apoptotic cells in the blastocysts of each group were examined. Data are based on at least 280 blastocyst samples from each group. **P < 0.01 and ***P < 0.001 *vs.* the control group.

Differential staining followed by cell counting was used to examine cell proliferation in blastocysts treated with 2.5, 5 or 10 μM 2-BP or left untreated for 24 h. Significantly lower cell numbers of ICM and TE were observed in 2-BP-treated blastocysts, compared to controls (Figure 2A). Furthermore, Annexin V staining revealed markedly higher Annexin V-positive/PI-negative (apoptotic) cells in the ICM and TE of treated blastocysts *vs.* controls (Figure 2B). These findings demonstrate that 2-BP induces significant apoptosis in the ICM and TE of mouse blastocysts, further supporting the theory that 2-BP impairs the implantation and developmental potential of blastocysts.

**Figure 3.**
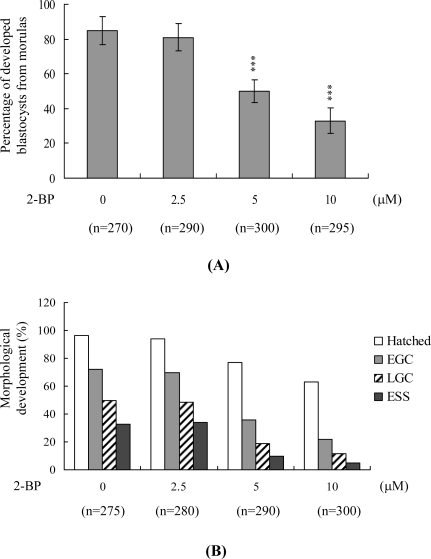
*In vitro* development of mouse embryos exposed to 2-BP at the blastocyst stage. (A) Mouse morulas were treated with or without 2-BP (2.5, 5 or 10 μM) for 24 h, and then cultured for an additional 24 h at 37 °C. Blastocysts were counted and percentages were calculated. (B) Mouse blastocysts were treated with or without 2-BP (2.5, 5 or 10 μM) for 24 h and observed in culture for 7 days post-treatment. Morphological assessments of hatched, early egg cylinder (EEC), late egg cylinder (LEC), and early somite stage (ESS) embryos were made according to established methods [29]. Values are presented as means ± SD of five to eight determinations. ***P < 0.001 *vs.* the control group.

The majority of untreated control morulas (85%) developed into blastocysts, whereas only 33–50% of morulas treated with 5–10 μM 2-BP developed into blastocysts under our experimental conditions (Figure 3A). To further establish the effects of 2-BP on implantation and postimplantation events *in vitro*, blastocysts were treated with 2.5, 5 or 10 μM 2-BP or left untreated (275–300 blastocysts, as indicated), and the rates of attachment to fibronectin-coated cultured dishes and subsequent development for 8 days in culture were assessed. 2-BP-pretreated blastocysts clearly displayed a lower incidence of attachment and postimplantation development (Figure 3B), formed two-layer ICM and ectoplacental cones at a lower rate, and showed fewer instances of embryos developing to the advanced egg cylinder stages (LEC and ESS stages) *vs.* untreated controls (Figure 3B). These results indicate that 2-BP affects *in vitro* implantation and the potential of blastocysts to develop features of postimplantation embryos.

**Figure 4.**
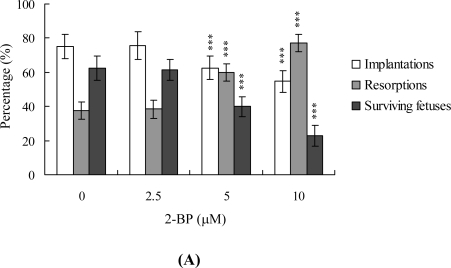

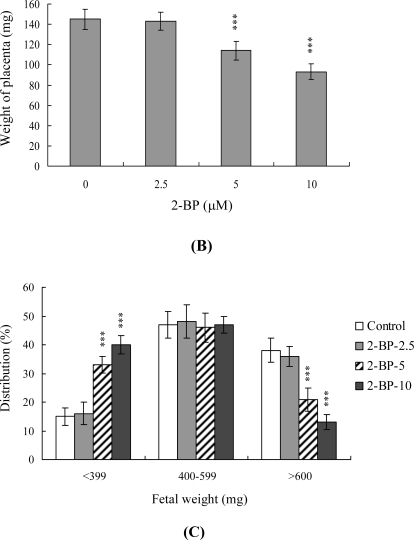
Effect of 2-BP treatment of mouse blastocysts on *in vivo* implantation, resorption, fetal survival and fetal weight by embryo transfer. (A) Mouse blastocysts were treated with or without 2-BP (2.5, 5 or 10 μM) for 24 h. Implantations, resorptions and surviving fetuses were analyzed as described in the Materials and Methods section. The percentage of implantations represents the number of implantations per number of transferred embryos × 100. The percentage of resorptions or surviving fetuses denotes the number of resorptions or surviving fetuses per number of implantations × 100. (B) The placental weights of 40 recipient mice were measured. (C) The weight distribution of surviving fetuses on day 18 post-coitus. Surviving fetuses (40–150 fetuses) were obtained by embryo transfer of control and 2-BP-pretreated blastocysts, as described in the Materials and Methods section (320 total blastocysts across 40 recipients). ***P < 0.001 *vs.* the 2-BP-free group.

To determine the effects of 2-BP on blastocyst development *in vivo*, we transferred mouse blastocysts (control and pretreated with 2-BP), and examined the uterine content at 13 days post-transfer (day 18 post-coitus). The implantation ratio in the 2-BP-pretreated group was lower than that in the untreated control group (Figure 4A). Moreover, the proportion of implanted embryos that failed to develop normally was significantly higher in groups pre-treated with 5 and 10 μM 2-BP (Figure 4A). Embryos that implanted but failed to develop were subsequently resorbed. 2-BP-pretreated mice displayed markedly higher early and late resorption rates than the untreated control group (Figure 4A). Furthermore, the placental weights of mice in the 2-BP-treated group were lower than those in the untreated group (Figure 4B), and fetal weight was lower in the 10 μM 2-BP-treated group, compared to controls (489 ± 51 mg *vs.* 607 ± 34 mg, respectively). Consistent with a previous study, recent experiments by our group show that 35–40% of fetuses weigh over 600 mg, and the average weight of total surviving fetuses is about 600 ± 12 mg in the untreated control group at day 18 of pregnancy in a mouse embryo transfer assay [20,27,28,30,31]. Fetal weight is an important gauge of developmental status. Accordingly, we used average fetal weight of the untreated control group as the key indicator to measure the development of 2-BP-treated blastocysts. Our results showed that only 13% of fetuses in the 10 μM 2-BP-pretreated group weighed over 600 mg (an important indicator of successful embryonic and fetal development), whereas 38% of control fetuses exceeded this threshold (Figure 4C). These findings collectively indicate that 2-BP exposure at the blastocyst stage reduces the potential of postimplantation development.

During the complex and precisely orchestrated process of embryonic development, chemical or physical injury can affect normal progression, leading to malformation or miscarriage of the embryo. Thus, it is important to establish the possible teratogenic effects of various chemical agents and environmental toxins. Exposure to 2-BP causes degeneration of germ cells via activation of apoptosis, and spermatogonia are the major target cells [2,32]. Interestingly, 2-BP induces apoptosis in germ cells through mitochondria-dependent apoptotic signaling molecules, Bcl-2/Bax, as well as Fas-FasL signaling pathway interactions [32]. In a previous animal study investigating the cytotoxic effects of 2-bromopropane (2-BP) on rat development, rats were exposed to inhaled 2-BP at concentrations of 0–1,000 ppm for 6 h per day, 7 days per week for 2 weeks prior to mating, during the mating period until copulation, and during days 0–19 of gestation. At 1,000 ppm, inhaled 2-BP significantly decreased the number of fetuses born, with exposure to this concentration of 2-BP causing fetal lethality during the post-implantation period [33]. However, the mechanisms regulating 2-BP cytotoxicity to embryonic development are not clear. In animal experiments, the cytotoxic effects of 2-BP on embryo development were observed only at the maximum concentration (1,000 ppm) and only after long exposure. We therefore used an *in vitro* assay system to assess the mechanisms by which 2-BP has cytotoxic effects on embryo development. To evaluate the possible cytotoxic effects and mechanisms of 2-BP on embryonic development, the present study used short-term higher concentrations of 2-BP treatment than those long-term exposure in animal models. In this model, mouse blastocysts cultured for 1–2 days in an incubator were co-incubated for 24 h with 2.5 to 10 μM 2-BP, concentrations higher than those that can be used in animal models. In the present study, preliminary time-course experiments showed that 2-BP triggers apoptosis in mouse blastocyst cells only after incubation for more than 12 h, which lasts for 24 h (data not shown). Based on this finding, we examined the effects of 2-BP on embryonic development by incubating blastocysts in medium containing 2.5 to 10 μM 2-BP for 24 h. Cell viability was decreased in mouse blastocysts owing to apoptosis (Figure 1). TUNEL staining revealed that treatment of mouse blastocysts with 2.5 to 10 μM 2-BP induced a 6.7- to 10.9-fold increase in apoptosis in a dose-dependent manner (Figure 1). Furthermore, dual differential and Annexin V staining disclosed 2-BP-induced cell loss in both the ICM and TE (Figure 2).

The TE arises from the trophoblast at the blastocyst stage and develops into a sphere of epithelial cells surrounding the ICM and blastocoel. These cells contribute to the placenta, and are required for development of the mammalian conceptus [34], signifying that a reduction in the TE cell lineage reduces implantation and embryonic viability [35,36]. In addition, previous studies found that a ~30% or more reduction in the number of cells in the ICM is associated with high risk of fetal loss or developmental injury, even in cases where implantation rate and TE cell numbers are normal [37]. Moreover, the ICM cell number is essential for proper implantation, and reduction in this cell lineage may decrease embryonic viability [35,36]. While apoptosis is responsible for eliminating unwanted cells during normal embryonic development, this process does not normally occur at the blastocyst stage [38,39]. Excessive apoptosis before or during the blastocyst stage is likely to delete important cell lineages, influencing embryonic development and potentially leading to miscarriage or embryonic malformation [40]. Thus, in view of the finding that 2-BP reduces the cell number and promotes apoptosis in both the ICM and TE of mouse blastocysts, we investigated the possibility that the compound causes implantation decrease, mortality and/or developmental delay of mouse embryos *in vitro* and *in vivo*. Our results show that 2-BP-treated blastocysts undergo decreased implantation and embryonic development and increased embryonic death *in vitro* and implantation *in vivo* (Figure 3 and Figure 4). Previous reports demonstrate that 2-BP induces apoptosis via interactions with mitochondria-dependent and Fas-FasL apoptotic pathways [32]. The regulatory mechanisms and pathways underlying the impact of 2-BP on embryonic development in our model are yet to be established.

## 3. Experimental Section

### 3.1. Chemicals

Pregnant mare’s serum gonadotropin (PMSG), Bovine serum albumin (BSA), sodium pyruvate and 2-bromopropane were purchased from Sigma (St. Louis, MO, USA). Human chorionic gonadotropin (hCG) was obtained from Serono (NV Organon Oss, the Netherlands). The TUNEL *in situ* cell death detection kit was obtained from Roche (Mannheim, Germany) and CMRL-1066 medium was from Gibco Life Technologies (Grand Island, NY, USA).

### 3.2. Collection of Mouse Morulas and Blastocysts

ICR mice were from National Laboratory Animal Center (Taiwan, ROC). This research was also approved by the Animal Research Ethics Board of Chung Yuan Christian University (Taiwan, ROC). All animals received humane care, as outlined in the Guidelines for Care and Use of Experimental Animals (Canadian Council on Animal Care, Ottawa, Canada, 1984). All mice were maintained on breeder chow (Harlan Teklad chow) with food and water available *ad libitum*. Housing was in standard 28 cm × 16 cm × 11 cm (height) polypropylene cages with wire-grid tops and kept under a 12 h day/12 h night regimen. Nulliparous females (6–8 weeks old) were superovulated by injection of 5 IU PMSG followed 48 hours later by injection of 5 IU hCG, and then mated overnight with a single fertile male of the same strain. The day a vaginal plug was found was defined as day 0 of gestation. Plug-positive females were separated for experimentation. Morulas were obtained by flushing the uterine tubes on the afternoon of gestation day 3, and blastocysts were obtained by flushing the uterine horn on day 4; in both cases the flushing solution consisted of CMRL-1066 culture medium containing 1 mM glutamine and 1 mM sodium pyruvate. The concentration of glucose in this medium was 5 mM. Expanded blastocysts from different females were pooled and randomly selected for experiments.

### 3.3. 2-BP Treatment and Tunel Assay

Blastocysts were incubated in medium containing the indicated concentrations of 2-BP for 24 h. For apoptosis detection, embryos were washed in 2-BP-free medium, fixed, permeabilized and subjected to TUNEL labeling using an *in situ* cell death detection kit (Roche Molecular Biochemicals, Mannheim, Germany) according to the manufacturer’s protocol. Photographic images were taken under brightfield illumination using a fluorescence microscope.

### 3.4. 2-BP Treatment and Cell Proliferation

Blastocysts were incubated with or without culture medium containing 2.5, 5 or 10 μM 2-BP. After 24h they were washed with 2-BP-free medium and dual differential staining was used to facilitate counting of cell numbers in the inner cell mass (ICM) and trophectoderm (TE) [35]. Blastocysts were incubated in 0.4% pronase in M_2_–BSA medium (M_2_ medium containing 0.1% bovine serum albumin) for removal of the zona pellucida. The denuded blastocysts were exposed to 1 mM trinitrobenzenesulphonic acid (TNBS) in BSA-free M_2_ medium containing 0.1% polyvinylpyrrolidone (PVP) at 4 °C for 30 min, and then washed with M_2_ medium [41]. The blastocysts were further treated with 30 μg/mL anti-dinitrophenol-BSA complex antibody in M_2_-BSA at 37 °C for 30 min, and then with M_2_ medium supplemented with 10% whole guinea-pig serum as a source of complement, along with 20 μg/mL bisbenzimide and 10 μg/mL propidium iodide (PI), at 37 °C for 30 min. The immunolysed blastocysts were gently transferred to slides and protected from light before observation. Under UV light excitation, the ICM cells (which take up bisbenzimidine but exclude PI) appeared blue, whereas the TE cells (which take up both fluorochromes) appeared orange-red. Since multinucleated cells are not common in preimplantation embryos [42], the number of nuclei was considered to represent an accurate measure of the cell number.

### 3.5. Annexin V Staining

Blastocysts were incubated in 0, 2.5, 5 or 10 μM 2-BP for 24 h, washed with 2-BP-free culture medium, and then stained using an Annexin V-FLUOS staining kit (Roche), according to the manufacturer’s instructions. Briefly, the blastocysts were incubated in M_2_-BSA for removal of the zona pellucida, washed with PBS plus 0.3% BSA, and then incubated for 60 min with a mixture of 100 μL binding buffer, 1 μL fluorescein isothiocyanate (FITC)-conjugated Annexin V and 1 μL PI. After incubation, the embryos were washed and photographed using a fluorescence microscope under fluorescent illumination. Cells staining Annexin V + /PI− were considered apoptotic, while those staining Annexin V + /PI + were considered necrotic.

### 3.6. Morphological Analysis of Embryonic Development

Blastocysts were cultured according to a modification of the previously reported method [43]. Briefly, embryos were cultured in 4-well multidishes at 37 °C. For group culture, four embryos were cultured per well. The basic medium consisted of CMRL-1066 supplemented with 1 mM glutamine and 1 mM sodium pyruvate plus 50 IU/mL penicillin and 50 mg/mL streptomycin (hereafter called culture medium). For treatments, the embryos were cultured with the indicated concentrations of 2-BP for 24 h in serum-free medium. Thereafter, the embryos were cultured for 3 days in culture medium supplemented with 20% fetal calf serum, and for 4 days in culture medium supplemented with 20% heated-inactivated human placental cord serum, for a total culture time of 8 days from the onset of treatment. Embryos were inspected daily under a phase-contrast dissecting microscope, and developmental stages were classified according to established methods [29]. Developmental parameters, such as hatching through the zona pellucida, attachment to the culture dish, trophoblastic outgrowth and differentiation of the embryo proper into early or late egg cylinders (germ layer stage) or primitive streak to early somite stage were recorded daily. To decrease observer bias, all the data were analyzed using the following criteria to differentiate *in vitro* stages of mouse embryos [29]. Implanted blastocyst was defined as the attachment and outgrowth of the blastocyst to the culture dish. An early egg cylinder (EEC) embryo was defined as an embryo that had reached stages 9 or 10 by day 4. A late egg cylinder (LEC) embryo was defined as an embryo that reached stages 11, 12 or 13 by day 6 of culture. An early somite stage (ESS) embryo was defined as an embryo that had reached stages 14 or 15 by day 8.

### 3.7. Blastocyst Development Following Embryo Transfer

To examine the ability of expanded blastocysts to implant and develop *in vivo*, the generated embryos were transferred to recipient mice. ICR females (white skin color) were mated with vasectomized males (C57BL/6J; black skin color; from National Laboratory Animal Center, Taiwan, ROC) to produce pseudopregnant dams as recipients for embryo transfer. To ensure that all fetuses in the pseudopregnant mice came from embryo transfer (white color) and not from fertilization by C57BL/6J (black color), we examined the skin color of the fetuses at day 18 post-coitus. To assess the impact of 2-BP on postimplantation growth *in vivo*, blastocysts were exposed to 0, 2.5, 5 and 10 μM 2-BP for 24 h, and then 8 embryos were transferred in parallel to the paired uterine horns of day 4 pseudopregnant mice. The surrogate mice were killed on day 18 post-coitus, and the frequency of implantation was calculated as the number of implantation sites per number of embryos transferred. The incidence rates of resorbed and surviving fetuses were calculated as the number of resorptions or surviving fetuses, respectively, per number of implantations. The weights of the surviving fetuses and placenta were measured immediately after dissection.

### 3.8. Statistics

The data were analyzed using one-way ANOVA and t-tests and are presented as the mean ± standard deviation, with significance at P < 0.05.

## 4. Conclusions

In summary, we have shown that 2-BP induces cellular apoptosis in both the ICM and TE of mouse blastocysts, leading to decreased implantation, embryonic development, and viability. Clearly, 2-BP is a potent injury risk factor for normal embryonic development. However, further studies are required to elucidate the mechanism(s) by which 2-BP affects embryonic development as well as the teratogenic actions and regulatory mechanisms of 2-BP in human embryogenesis.
